# Electrical Characteristics of Solution-Based Thin-Film Transistors with a Zinc-Tin Oxide/Carbon Nanotube Stacked Nanocomposite Active Layer

**DOI:** 10.3390/nano15010022

**Published:** 2024-12-27

**Authors:** Yong-Jae Kim, Woon-Seop Choi

**Affiliations:** Department of Semiconductor Engineering, Hoseo University, Asan 31499, Republic of Korea

**Keywords:** oxide TFT, semiconductor, carbon nanotube, nanocomposite

## Abstract

A stacked nanocomposite zinc-tin oxide/single-walled carbon nanotubes (ZTO/SWNTs) active layer was fabricated for thin-film transistors (TFTs) as an alternative to the conventional single-layer structure of mixed ZTO and SWNTs. The stacked nanocomposite of the solution-processed TFTs was prepared using UV/O_3_ treatment and multiple annealing steps for each layer. The electrical properties of the stacked device were superior to those of the single-layer TFT. The ZTO/SWNT TFT, fabricated using a stacked structure with ZTO on the top and SWNT at the bottom layer, showed a significant improvement in the field-effect mobility of 15.37 ${\mathrm{cm}}^{2}/V\cdot s$ (factor of three increase) and an I_on_/I_off_ current ratio of 8.83 × $10^{8}$ with improved hysteresis. This outcome was attributed to the surface treatment and multiple annealing of the selected active layer, resulting in improved contact and a dense structure. This was also attributed to the controlled dispersion of SWNT, as electron migration paths without dispersants. This study suggests the potential expansion of applications, such as flexible electronics and low-cost fabrication of TFTs.

## 1. Introduction

Thin-film transistors (TFTs) are used widely in various fields, such as flat panel displays, flexible electronics, and sensors. The most common channel materials for TFTs are amorphous silicon (a-Si) and polycrystalline silicon (poly-Si). Among them, a-Si TFTs have low cost and high uniformity but generally have low mobility of less than 2 ${\mathrm{cm}}^{2}/V\cdot s$ and poor electrical stability [1]. Poly-Si TFTs have higher mobility than a-Si TFTs, but their widespread use is limited by the complex fabrication process, high cost, restricted mechanical flexibility, and non-uniform device characteristics over large areas [2].

Oxide TFTs, such as indium oxide (IO), zinc oxide (ZnO), and indium-gallium-zinc oxide (IGZO), have been studied extensively because of their low processing cost, simple packaging, and better compatibility with flexible substrates compared to the two technologies mentioned above [3,4,5]. On the other hand, their relatively low mobility still fails to meet the requirements of future high-performance display applications [6].

Single-walled carbon nanotubes (SWNTs) have attracted significant attention as a promising candidate for the high-performance channels of TFTs owing to their excellent mechanical and electrical properties [7,8]. Researchers have used SWNTs as the active channel materials in organic semiconductors [9] or have combined SWNTs with indium-zinc oxide (IZO) or amorphous-IGZO to enhance the electrical characteristics of TFTs [10,11]. These studies reported substantial improvements in the mobility and electrical properties of composite TFTs containing SWNTs within a mixed type. Nevertheless, avoiding the use of indium, a rare metal essential for the production of electrically activated high-performance solution-based oxide semiconductors, remains a critical challenge in the pursuit of both cost efficiency and improved device performance. Many studies have overlooked cost considerations, often relying on indium- and gallium-based materials.

Among the many semiconductors in oxide TFTs, zinc-tin oxide (ZTO) has attracted attention because it does not contain expensive rare elements, such as indium and gallium. Furthermore, ZTO is a promising material for future oxide applications because of its wide bandgap, low cost, and non-toxicity. ZTO TFTs can be fabricated using various methods, including solution processing, ink-jet printing, and electrohydrodynamic jet printing [12,13,14]. In particular, solution processing is a major trend in TFT manufacturing because it is relatively simple and cost-effective.

Despite not requiring high-vacuum and high-pressure operating environments, solution-processed TFTs still have some defects in the transfer characteristics and reliability. Solution-processed TFTs exhibit relatively low electrical performance, and it is difficult to completely avoid substantial uncertainties and the attachment of external impurities on the TFT surface [5].

Previous research on TFTs faced challenges in uniformly dispersing SWNTs when they were mixed directly into the device. While this approach improved electrical properties compared to devices without SWNTs, the performance enhancement was limited, and significant improvements were hard to achieve. Despite previous studies using a single structure of a semiconductor and SWNTs as the active layer of TFTs, there are few reports of different structural configurations of ZTO and SWNTs as the active layer.

This study prepared ZTO/SWNT-stacked active layers with different geometric nanocomposite structures of SWNT and ZTO in semiconductors for TFTs. A hybrid stacked structure was introduced with uniformly dispersed SWNTs through surface treatments and repeated annealing steps for each layer. This method ensured better distribution of SWNTs without using dispersants, leading to significantly improved electrical performances. The ZTO/SWNT TFTs with the hybrid stacked layer exhibited higher transparency and outperformed the electrical properties of single-layer devices. Therefore, a stacked nanocomposite ZTO/SWNT active layer may be an alternating structure for TFT fabrication and electronic applications.

## 2. Experimental

Zinc acetate dihydrate and tin chloride were mixed in 2-methoxyethanol as a stabilized sol-gel precursor solution for the active layer. The concentration of the metal precursors was 0.3 M, with equal molar ratios of Zn and Sn. The solution was stirred for 24 h at 50 °C. The SWNTs, [single-walled nanotubes > 90 wt.%, average diameter: 1.1 nm, length: 5–30 μm, US Research Nanomaterials, Inc., Houston, TX, USA], were dispersed in ethanol as a dispersing solvent at 20 °C for 24 h and followed by ultrasonication at 28 kHz for one hour at R.T. The SWNT concentration was calculated based on the mass ratio of SWNTs to the Zn and Sn precursors, ranging from 0.01–0.07 wt.%.

The following presents an example of fabricating the stacked structure of ZTO at the bottom and SWNT on the top (sample C). A 0.3 M ZTO solution was filtered through a 5 μm filter and spin-coated at 5500 rpm for 30 s on the substrate, which underwent UV/$O_{3}\;$surface treatment to improve wettability. The resulting film was thermally treated at 150 °C for 30 min in air to evaporate the solvent, followed by another UV/$O_{3}\;$surface treatment. A SWNT solution with various concentrations (0.01–0.07 wt.%) was dispersed on top of the ZTO thin film via ultra-sonication and spin-coated at 5500 rpm for 30 s. The film was then annealed by heating at 550 °C for one hour and 20 min to form a metal-oxide framework. The thickness of the active layer was 30 nm. For the fabrication of the reverse structure of SWNT at the bottom and ZTO on top (sample D), the substrate process was also performed using the same methodology.

**Figure 1:**
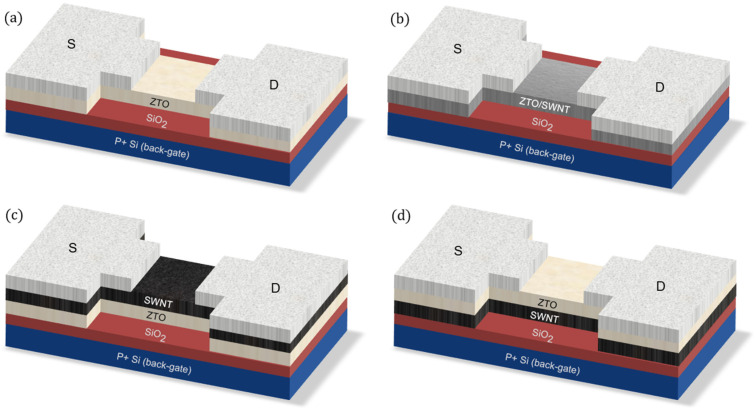
Schematic cross-section views of the bottom gate staggered structure for (**a**) sample A (ZTO only), (**b**) sample B (ZTO/SWNTs mixed), (**c**) sample C (Top SWNT bottom ZTO), (**d**) sample D (Top ZTO bottom SWNT). (S: Source, D: Drain).

Aluminum as a source and drain was deposited at a rate of 3 Å/s with a shadow mask on the top of a thermally-annealed ZTO layer on the substrate using a thermal evaporator to have a thickness of 1100 Å. No additional heat treatment was performed. The TFT device was fabricated in a bottom-gate structure with 1500 µm/100 µm of width/length of the active layer, as shown in the schematic cross-sectional view in Figure 1. Highly doped silicon (Si) was used as the back gate, and 300 nm of thermally-grown silicon dioxide (${\mathrm{SiO}}_{2}$) was used as the gate insulator. The current-voltage (I-V) characteristics of the ZTO/SWNT TFTs were measured at room temperature in a dark box using a semiconductor parameter analyzer (Keithley 4200A-SCS, Cleveland, OH, USA). The structural properties of the solution-processed ZTO/SWNTs thin film were examined using optical microscopy (Olympus-BX51M, Tokyo, Japan) and scanning electron microscopy (SEM, TESCAN:MAIA3, Brno, Czech Republic).

## 3. Results and Discussion

In the process of preparing the ZTO/SWNT solution, we minimized the use of chemicals without dispersants and chose less harmful materials. The fabrication process did not rely on photolithography, avoiding the use of energy-intensive equipment and hazardous chemicals. This simplified the manufacturing process, reduced waste, and minimized the environmental footprint.

Stacked nanocomposite structured active layers are proposed to improve the electrical characteristics of solution-processed ZTO TFTs, as shown in Figure 1: (a) ZTO TFT active layer without SWNTs {Figure 1a, sample A}, (b) active layer with a mixture of ZTO and SWNTs {Figure 1b, sample B}, (c) stacked structure with a SWNTs film on the top of a ZTO film {Figure 1c, sample C}, and (d) stacked structure with a SWNTs film at the bottom and a thermally-treated ZTO film on the top {Figure 1d, sample D}.

For effectively stacked structures, unlike the previous method of mixing SWNTs into the ZTO solution, the substrate of the selected layer underwent a UV/O_3_ treatment and was coated with a ZTO solution and SWNTs solution. Subsequently, additional UV surface treatment and annealing were performed. Various concentrations (0.01–0.07 wt.%) of SWNT solutions were used during this process. The improved adhesion between the layers of ZTO and SWNTs was attributed to the surface treatment and annealing approaches carried out in the selected layer. In terms of the coating sequence, various coating conditions were explored, including coating ZTO first and then SWNTs, as well as coating SWNTs first and then ZTO, which is different from the conventional coating of ZTO/SWNTs mixture. This allowed the coating characteristics and film quality, leading to the fabrication of high-quality semiconductor layers for TFTs.

**Figure 2:**
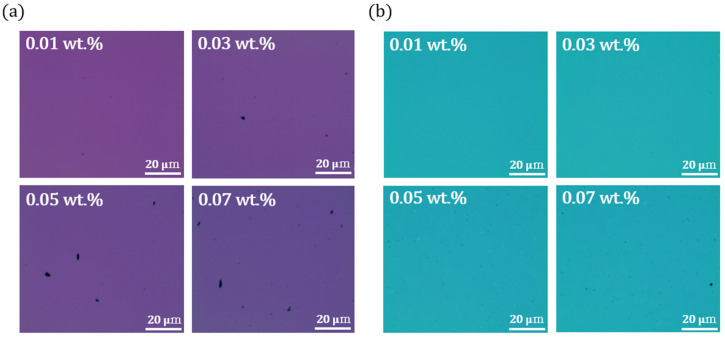
(**a**) Optical microscopy images of annealed sample C at various SWNT concentrations (0.01–0.07 wt.%), (**b**) Optical microscopy images of annealed sample D at various SWNT concentrations (0.01–0.07 wt.%).

Figure 2 presents optical microscopy images of the stacked structure of SWNTs/ZTO (sample C) and the ZTO/SWNTs (sample D) structure. The optical microscopy images showed that an increase in SWNT content (0.01–0.07 wt.%) in the ZTO precursor solution leads to an increase in the SWNT amount on the substrate after the annealing process. These results emphasize the importance of controlling the dispersion state of SWNTs in the solution during the experiment without any surface-active agents and the significance of the annealing process in obtaining high-quality data.

The annealing process is crucial in properly drying the ZTO/SWNTs solution and stably fixing the SWNTs. When the solvent evaporates, the SWNTs can agglomerate and form clusters if they are not well dispersed, which may affect the characteristics of the film with concentrations of the SWNTs. Therefore, it is essential to monitor the dispersion state of the SWNTs using optical microscopy after annealing to ensure the reliability of the experimental results.

**Figure 3:**
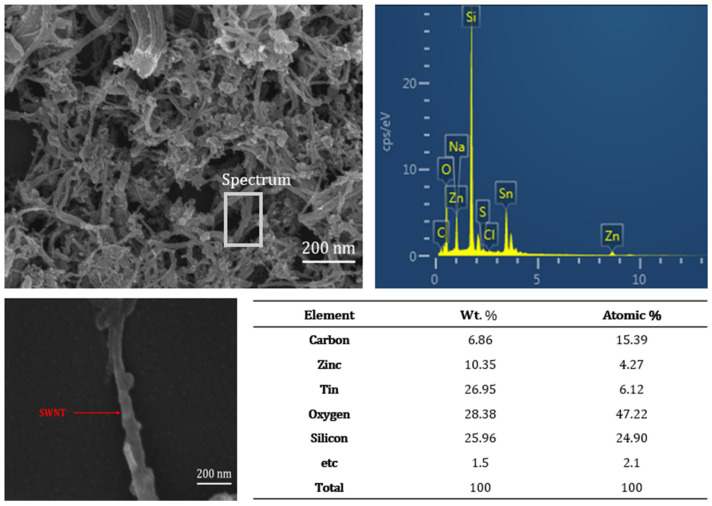
SEM images, mapping, and EDX elements of ZTO/SWNT film with 0.07 wt.% SWNTs concentration with a scale bar of 200 nm.

Figure 3 presents SEM images showing the presence of SWNTs within the spin-coated ZTO/SWNTs nanocomposite thin-film transistor. The spin-coated ZTO/SWNTs nanocomposite thin-film exhibited a dense and continuous structure, indicating the strong connection between ZTO and the SWNTs structure, facilitating excellent charge transport between the source and drain electrodes. EDX, mapping to the SEM image, and element table were shown in Figure 3 to confirm the uniform distribution of the ZTO component and SWNTs throughout the sample.

There may be some physical and chemical interactions at the ZTO/SWNT interface. In the stacked ZTO/SWNT devices, repeated surface treatments and annealing processes are used to improve the surface properties of ZTO with SWNTs. This optimization can significantly reduce contact resistance and improve the oxygen vacancies and other oxygen-related defects. While weak interactions may exist between ZTO/SWNTs nanocomposite, the various treatment processes may promote the formation of percolation between ZTO and SWNTs to provide conductive paths, which enhance the charge transport pathways and improve device performance.

**Figure 4:**
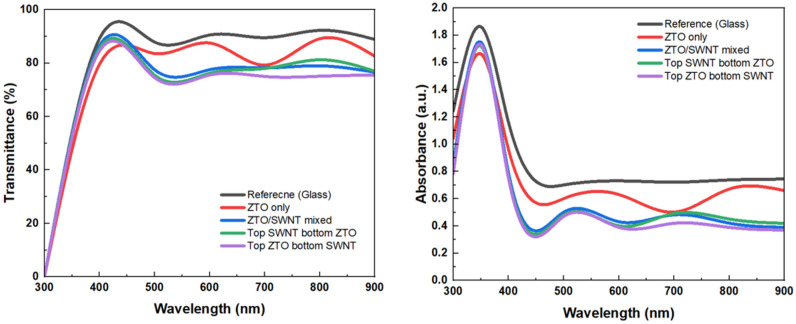
Transmittance and absorption spectra of ZTO/SWNT thin films on glass with different SWNT concentrations.

Stacked active layers in different geometries may alter the thin film properties with SWNT concentrations. Figure 4 presents the optical transmittance and absorption spectra of ZTO/SWNT nanocomposite on glass with different SWNT concentrations in the wavelength range of 300–1000 nm. Although the transmittance of ZTO/SWNT thin film was less than that of ZTO thin film without SWNT because of the light scattering of SWNTs, all films showed good transmittances above 80% in the visible-light region. Despite the inclusion of SWNTs, the optical transmittance remains largely unaffected, aligning with the key optical requirements for transparent devices. The optical absorption coefficient was calculated from the transmittance, and the optical band gap (*E_g_*) was calculated using the following equation

$$\alpha (hv)={\left(hv-E_{g}\right)}^{1/2}$$

where *h* is the Planck constant and *v* is the frequency of the incident photon. The *E_g_* at different SWNT concentrations increased slightly from 3.63 eV without SWNT to 3.73 eV with 0.07 wt.% SWNTs. This increase in the band gap may be explained by the increasing the carrier concentration [15].

**Figure 5:**
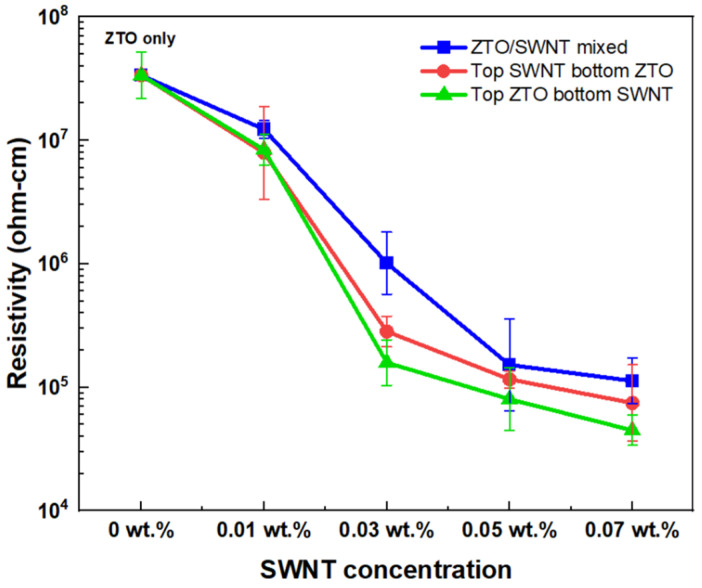
Resistivity of ZTO/SWNT thin films with different SWNT concentrations.

Figure 5 shows the resistance changes in the single-layer ZTO/SWNTs TFT (ZTO/SWNT mixed active layer) and the stacked ZTO/SWNTs TFT with various SWNTs concentrations. The resistance of the single-layer ZTO/SWNTs film was approximately 7.42 × $10^{7}$ Ω-cm, which were measured after depositing the source and drain electrodes. In the stacked structure, the SWNTs/ZTO film with the ZTO at the bottom (sample C) exhibited a resistance of 6.12 × $10^{4}$ Ω-cm, while the ZTO/SWNTs film with the SWNTs at the bottom (Sample D) showed a resistance of 2.51 × $10^{4}$ Ω-cm. The stacked nanocomposite structure with SWNTs at the bottom (sample D) exhibited the lowest resistance among the samples, particularly at the highest SWNTs concentration of 0.07 wt.%, which may be expected for the best device performance. Interestingly, as the concentration of metallic SWNTs increased, the resistance of the device decreased, and the resistance of the nanocomposite structure TFT was lower than that of the conventional single-layer TFT (ZTO/SWNT mixed active layer). This analysis suggests that metallic SWNTs facilitate electron transport within the device and that the stacked nanocomposite structure may be more effective for enhancing the device’s performance than the general mixed-layer approach.

**Figure 6:**
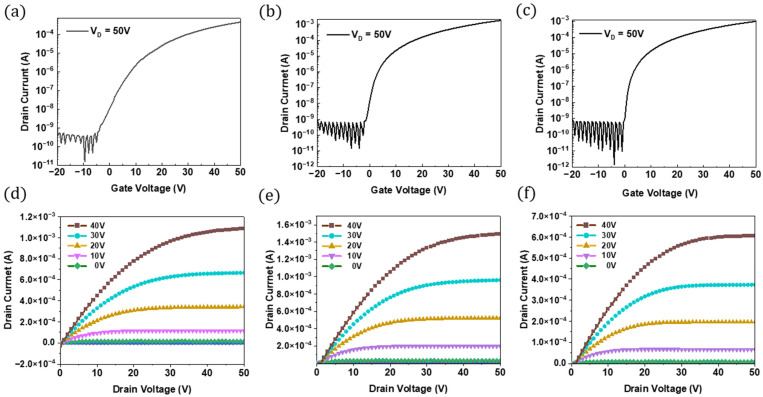
Transfer characteristics and output characteristics of a SWNT/ZTO TFTs with 0.07 wt.% SWNT concentration. (**a**,**d**) sample A (ZTO only) (**b**,**e**) sample C (Top SWNT bottom ZTO) (**c**,**f**) sample D (Top ZTO bottom SWNT).

Figure 6a–c shows the drain current (I_d_) versus gate voltage (V_g_) (I_d_-V_g_) transfer characteristics of the ZTO/SWNTs TFTs with single-layer and stacked channel structures at a gate voltage of 50 V. The electrical characteristics, such as effective mobility, threshold voltage, l_on_/l_off_ current ratio, and subthreshold swing, were derived through a linear fit of the square root of V_g_ versus I_d_ plot using the standard saturation equation. Figure 5 shows the electrical characteristics for various drain voltages (V_ds_) for the ZTO/SWNT devices with 0.07 wt.% SWNT concentration (samples A, C, and D). All the devices containing SWNTs exhibited more pronounced TFT characteristics than devices without SWNTs.

Figure 6d–f shows the drain current versus drain voltage (I_d_-V_d_) output characteristics of ZTO/SWNTs TFTs with single-layer and stacked channel structures at various gate voltages. These correspond to samples A, C, and D, respectively. The ZTO/SWNT semiconductors underwent high-temperature annealing to improve contact between the SWNTs and the ZTO layer, reducing the contact resistance. After annealing, the surface of the ZTO/SWNT thin film became smoother and more uniform, resulting in a reduced contact resistance between ZTO and SWNTs. In addition, samples containing SWNTs exhibited saturation characteristics at relatively low gate voltages. The increased carrier density of the ZTO/SWNTs, as described in the band gap calculation, enhanced the electrical properties of the device through high-temperature annealing, which is similar to a previous study of a-IGZO with the SWNT electrode devices [16].

**Figure 7:**
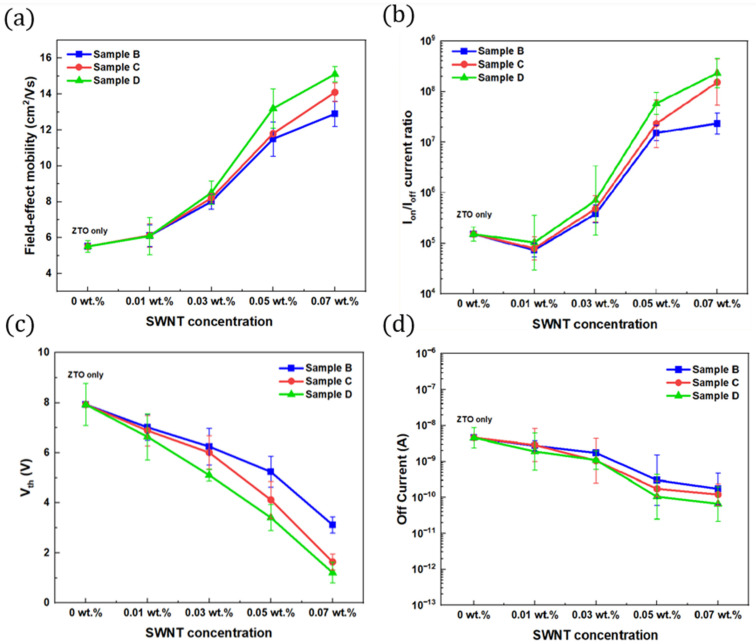
(**a**) Field-effect mobility vs. SWNT concentration. (**b**) I_on_/I_off_ current ratio vs. SWNTs concentration. (**c**) Vth vs. SWNT concentrations. (**d**) Off current vs. SWNT concentrations. All for the ZTO/SWNT nanocomposite TFTs.

Table 1 lists the device parameters. The saturation field-effect mobilities (μ_sat_) for samples A, B, C, and D were 5.51 ${\mathrm{cm}}^{2}/V\cdot s$, 11.14 ${\mathrm{cm}}^{2}/V\cdot s$, 13.91 ${\mathrm{cm}}^{2}/V\cdot s$, and 15.37 ${\mathrm{cm}}^{2}/V\cdot s$, respectively. The mobility increased dramatically from approximately 5.51 ${\mathrm{cm}}^{2}/V\cdot s$ for the ZTO TFT without SWNTs to approximately 15.37 ${\mathrm{cm}}^{2}/V\cdot s$ for the TFT with a 0.07 wt.% SWNT concentration. Figure 7a shows the mobility data for TFTs with different structures and various SWNT concentrations. The conductive SWNTs provide high-speed tracks for carrier transport, allowing the electrons flowing through the SWNTs to move faster than those flowing through the regular channel. As a result, carrier mobility improved significantly.

The I_on_/I_off_ current ratio increased from ~$10^{4}$ for the ZTO TFT without SWNTs to ~$10^{8}$ for the TFT with 0.07 wt.% SWNTs, as shown in Figure 7b. The I_on_/I_off_ current ratio of the TFTs increased as the SWNT concentrations increased. The stacked nanocomposite structure TFT (sample D) with 0.07 wt.% of SWNTs showed the highest ratio of 8.83 × $10^{8}$. The subthreshold slopes for samples A, B, C, and D were 3.11 V/decade, 3.15 V/decade, 1.15 V/decade, and 1.01 V/decade, respectively. It decreased to 1.01 V/decade for the TFT with 0.07 wt.% SWNTs.

The threshold voltages (Vth) for samples A, B, C, and D were 7.92 V, 3.12 V, 1.64 V, and 1.21 V, respectively. The Vth of the TFTs decreased as SWNT concentration increased, as shown in Figure 7c. The average Vth of the devices improved from 7.92 V to 1.21 V, including SWNTs. Compared to the ZTO TFT, the ZTO/SWNTs nanocomposite TFTs with SWNTs showed a decrease in Vth. Moreover, stacked nanocomposite structures demonstrated much lower Vth values than the traditional mixed structure.

The mechanism for improving the threshold voltage by adding SWNTs was attributed to metallic SWNTs on the channel surface, which partially provide carriers during conduction. In addition, the inclusion of SWNTs helps reduce the Vth by supplying some of the required carriers through the metallic SWNTs on the active channel surface, lowering the gate voltage needed for the active channel [17]. The off current also improved as the SWNT concentration increased, as shown in Figure 7d. Moreover, the subthreshold slope of the device decreased from 3.11 to 1.13 V/dec.

The hybrid stacked TFTs outperformed the single-layer TFTs in terms of electrical properties with improved stability [18]. Furthermore, the UV surface treatment and multiple annealing of the selected active layer optimized the crystalline structure and reduced the defect density. For bottom-gate SWNT TFTs, where the SWNT channel is formed near the interface between the gate insulator and the active layer, UV surface treatment and annealing were most effective in enhancing device characteristics. The proposed multi-layer stacking and the selective UV/O_3_ and annealing treatment methods could provide the desired electrical properties. Meanwhile, the non-annealed samples showed significantly inferior electrical performance to the annealed samples due to an increase in interface trap density and the influence of residual chemicals.

**Figure 8:**
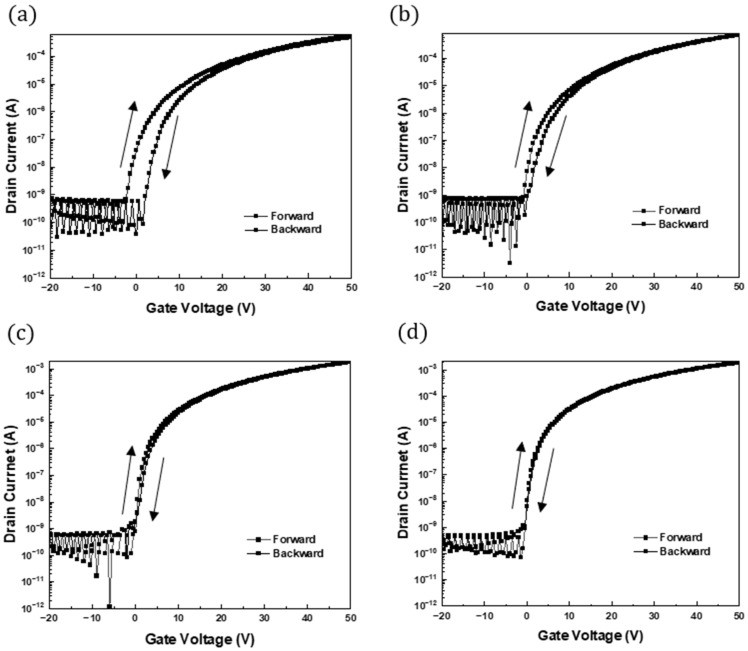
Hysteresis characteristics of a ZTO only and ZTO/SWNTs (0.07 wt.%) TFTs. (**a**) sample A (ZTO only), (**b**) sample B (ZTO/SWNTs mixed), (**c**) sample C (Top SWNTs bottom ZTO), (**d**) sample D (Top ZTO bottom SWNTs).

Figure 8 shows the hysteresis of ZTO TFTs containing 0.07 wt. SWNTs. When the gate voltage was scanned to 50 V, a clockwise hysteresis loop was observed in the transfer curve, as shown in Figure 8a. Figure 8b presents the hysteresis results of the most optimal 0.07 wt.% SWNT mixed ZTO TFT (sample B) among the SWNT concentrations assessed in this study. Figure 8c presents the hysteresis results of TFTs with a stack structure, where ZTO is at the bottom and SWNTs are at the top (sample C). Figure 8d shows the hysteresis results of TFTs with the opposite configuration (top ZTO and bottom SWNTs, sample D). The gate voltage shifts associated with the hysteresis for Samples B, C, and D were approximately 1.68 V, 0.41 V, and 0.15 V, respectively. The ZTO/SWNTs TFTs exhibited much-reduced hysteresis compared to TFTs without SWNTs. Furthermore, the hysteresis of the TFTs fabricated with the layered stack structure was lower than that of the TFTs using general mixed ZTO and SWNT systems.

The clockwise hysteresis observed in the transfer characteristics stems primarily from interface traps between the SWNTs and the gate dielectric. Furthermore, it is caused by charge trapping introduced from the gate dielectric and absorption of moisture and oxygen molecules on the ZTO/SWNT network surface. The existence of clockwise hysteresis aligns with electron trapping near the oxide semiconductor-gate dielectric interface or within the oxide semiconductor channel layer [3,19]. Nevertheless, this clockwise hysteresis is not associated with an instability mechanism linked to ion migration (either negative or positive) within the gate dielectric, which is considered irreversible [20]. The device fabricated using a stacked nanocomposite structure, ZTO/SWNTs (sample D), exhibited the smallest hysteresis, almost hysteresis-free operation, as shown in Figure 8d. Therefore, the TFT devices fabricated using stacked structures had the potential to operate without hysteresis. Devices subjected to multiple annealing processes reduce moisture absorption on the SWNT network, reducing charge injection into the device area and decreasing hysteresis. The annealing process helps compensate for oxygen vacancies in the ZTO/SWNT layer, which act as trap sites and can disrupt device stability. This corresponds to reports indicating that the adsorption of water and oxygen molecules in atmospheric environments plays a crucial role in reducing hysteresis in SWNT TFT devices [20]. TFT devices fabricated using a stacked nanocomposite structure could operate without hysteresis, exhibiting excellent properties.

**Figure 9:**
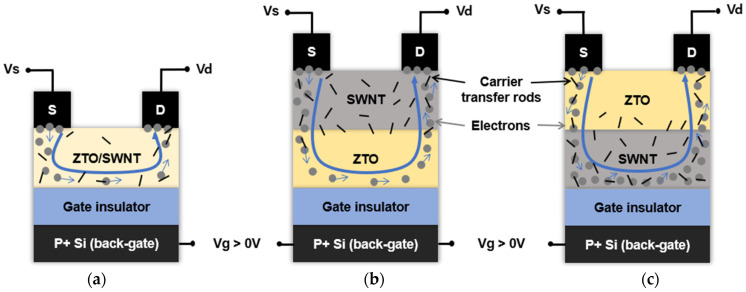
Approach to a solution-based ZTO through a blend of SWNT as carrier transport rods to increase the electrical performance, (**a**) SWNT mixed ZTO TFT (**b**) top SWNTs bottom ZTO TFT (**c**) top ZTO bottom SWNTs TFT. (S: Source, D: Drain).

Figure 9 presents the approach to explain a potential mechanism for enhancing the electrical performance of ZTO TFTs by incorporating SWNTs as carrier transport rods in the active layer, as described above. The electrical properties of the ZTO/SWNT TFTs are strongly influenced by the density and aggregation structure of the SWNTs, as shown in Figure 9. Compared to the random distribution of SWNT in the thin films fabricated by simple dispersion of the conventional ZTO/SWNT thin films (sample A and Figure 9a), the stacked nanostructure devices fabricated with a hybrid layer of ZTO and SWNT could provide more SWNT junctions in the active layer (sample C and D, and Figure 9b,c). In ZTO/SWNT mixed devices, the distance between SWNTs is too large to form effective electron transport pathways. The density of SWNTs increases significantly when stacking layers with SWNTs packed closer and aligned near gate dielectric in devices where ZTO is on top, and SWNT is underneath (Figure 9c). Hence, this structure provides more favorable conditions as carrier transport rods for enhancing the electrical performance of the device. The proposed stacked active layer for TFTs is an effective method of improving the electrical properties.

## 4. Conclusions

This paper proposed a novel structure of the nanocomposite ZTO/SWNT TFTs for enhancing electrical parameters. Various configurations of ZTO/SWNT stacked structures were analyzed to achieve optimal performance by altering the placement of SWNT within the channel region. Three different channel configurations (ZTO/SWNT blend, ZTO at the bottom with SWNT on top, and ZTO on top with SWNT at the bottom) were prepared. The TFT with SWNT at the bottom showed a three-fold increase in mobility (from 5.51 to 15.37$\;{\mathrm{cm}}^{2}/V\cdot s$) and l_on_/l_off_ current ratio over $10^{8}$ with much-improved hysteresis. In addition, the ZTO/SWNT stacked nanocomposite structure exhibited superior performance to the ZTO/SWNT blend structure. Overall, ZTO/SWNT nanocomposite TFTs are a promising TFT configuration that can achieve improved device performances and integrate nanomaterials for various electronic applications. The potential for large-area integration makes it a promising candidate for next-generation display technologies with high performance and cost-effective fabrication.

## Figures and Tables

**Table 1:** Electrical Characteristics of Fabricated ZTO/SWNT TFTs.

	$\mu_{\mathrm{sat}}\;({\mathrm{cm}}^{2}/V\cdot s)$	$I_{\mathrm{on}/\mathrm{off}}$	$V_{\mathrm{th}}\left(V\right)$	SS (V/Decade)
**Sample A** **(ZTO TFT without SWNT)**	5.51	$3.42\;\times \;10^{4}$	7.92	3.11
**Sample B** **(ZTO TFT blend 0.07 wt.% SWNT)**	11.14	$1.45\;\times \;10^{7}$	3.12	3.15
**Sample C** **(Top 0.07 wt.% SWNT bottom ZTO)**	13.91	$5.41\;\times \;10^{8}$	1.64	1.15
**Sample D** **(Top ZTO bottom 0.07 wt.% SWNT)**	15.37	$8.83\;\times \;10^{8}$	1.21	1.01

## Data Availability

Data are contained within the article.

## References

[1] Fortunato E.M., Barquinha P.M., Pimentel A.C.M.B.G., Goncalves A.M., Marques A.J., Pereira L.M., Martins R.F. (2005). Fully transparent ZnO thin-film transistor produced at room temperature. Adv. Mater..

[2] Lee C.H., Striakhilev D., Tao S., Nathan A. (2005). Top-gate TFTs using 13.56 MHz PECVD microcrystalline silicon. IEEE Electron. Device Lett..

[3] Lee J.S., Kwack Y.J., Choi W.S. (2013). Inkjet-printed In_2_O_3_ thin-film transistor below 200 °C. ACS Appl. Mater. Interfaces.

[4] Li S., Chen X., Liu L., Zeng Z., Chang S., Wang H., Wu H., Long S., Liu C., Liu C. (2022). Micron channel length ZnO thin film transistors using bilayer electrodes. J. Colloid Interface Sci..

[5] Kamiya T., Nomura K., Hosono H. (2010). Present status of amorphous In–Ga–Zn–O thin-film transistors. Sci. Tech. Adv. Mater..

[6] Jeon Y., Lee D., Yoo H. (2022). Recent advances in metal-oxide thin-film transistors: Flexible/stretchable devices, integrated circuits, biosensors, and neuromorphic applications. Coatings.

[7] Han S.J., Tang J., Kumar B., Falk A., Farmer D., Tulevski G., Jenkins K., Afzali A., Oida S., Ott J. (2017). High-speed logic integrated circuits with solution-processed self-assembled carbon nanotubes. Nat. Nanotechnol..

[8] Tang J., Cao Q., Tulevski G., Jenkins K.A., Nela L., Farmer D.B., Han S.J. (2018). Flexible CMOS integrated circuits based on carbon nanotubes with sub-10 ns stage delays. Nat. Electron..

[9] Bo X.Z., Tassi N.G., Lee C.Y., Strano M.S., Nuckolls C., Blanchet G.B. (2005). Pentacene-carbon nanotubes: Semiconducting assemblies for thin-film transistor applications. Appl. Phys. Lett..

[10] Liu X., Wang C., Cai B., Xiao X., Guo S., Fan Z., Liao L. (2012). Rational design of amorphous indium zinc oxide/carbon nanotube hybrid film for unique performance transistors. Nano Lett..

[11] Chen H., Cao Y., Zhang J., Zhou C. (2014). Large-scale complementary macroelectronics using hybrid integration of carbon nanotubes and IGZO thin-film transistors. Nat. Commun..

[12] Lee Y.G., Choi W.S. (2014). Electrohydrodynamic jet-printed zinc–tin oxide TFTs and their bias stability. ACS Appl. Mater. Interfaces.

[13] Kim H., Kwack Y.J., Yun E.J., Choi W.S. (2016). A mixed solution-processed gate dielectric for zinc-tin oxide thin-film transistor and its MIS capacitance. Sci. Rep..

[14] Sykora B., Wang D., von Seggern H. (2016). Multiple ink-jet printed zinc tin oxide layers with improved TFT performance. Appl. Phys. Lett..

[15] Burstein E. (1954). Anomalous optical absorption limit in InSb. Phys. Rev..

[16] Zhu K., Wen L., Zhang M. (2017). A carbon nanotube electrode a-IGZO-TFT. IEEE J. Electron. Dev. Soc..

[17] Wu Y., Zhang M., Xiao X., Zhang S. Indium gallium zinc oxide-Carbon nanotube composite thin film transistor. Proceedings of the 2014 IEEE International Conference on Electron Devices and Solid-State Circuits.

[18] Guo H.B., Shan F., Kim H.S., Lee J.Y., Kim N., Zhao Y., Kim S.J. (2020). Amorphous oxide thin-film transistors and inverters enabled by solution-processed multi-layers as active channels. AIP Adv..

[19] Wager J.F., Keszler D.A., Presley R.E. (2008). Transparent Electronics.

[20] Yi X., Yao Y., Zhao Z., Yu P., Zou H., Luo D. Study on Transfer Characteristic Hysteresis of Carbon Nanotube Thin Film Transistor. Proceedings of the 2023 5th International Conference on Electrical Engineering and Control Technologies (CEECT).

